# Current Assessment of Weight, Dietary and Physical Activity Behaviors among Middle and High School Students in Shanghai, China—A 2019 Cross-Sectional Study

**DOI:** 10.3390/nu13124331

**Published:** 2021-11-30

**Authors:** Jingfen Zhu, Yinliang Tan, Weiyi Lu, Yaping He, Zhiping Yu

**Affiliations:** 1 School of Public Health, Shanghai Jiao Tong University, Shanghai 200025, China; zhujingfenjt@shsmu.edu.cn (J.Z.); MelodyBeata@sjtu.edu.cn (Y.T.); vivienlu999@shsmu.edu.cn (W.L.); hypcyr@sina.com (Y.H.); 2 Department of Nutrition and Dietetics, University of North Florida, Jacksonville, FL 32224, USA

**Keywords:** obesity, overweight, adolescents, dietary, physical activity, sedentary, Chinese

## Abstract

Poor nutrition or insufficient physical activity (PA) are risk factors for obesity and chronic diseases. This 2019 cross-sectional study from the school health survey examined the dietary and PA behaviors of Chinese adolescents. A total of 12,860 adolescents aged 11–18 participated through multistage and stratified cluster random sampling. A questionnaire collected data on weight, PA, sedentary lifestyle, and eating habits. Unhealthy behaviors were identified and summed up for each behavior. Participants were then classified into high and low amounts of risk behaviors. Weight status was defined using Body Mass Index (BMI) cutoff points for Chinese individuals aged 6–18. Multinomial logistic regression was used to assess effects of lifestyle behaviors on weight status. The prevalence of overweight and obesity was 22.3% among all participants (30.6% in boys, 13.2% in girls). Females engaged in more risk physical activities (4.12 vs. 3.80, *p* < 0.05), while males engaged in more risk dietary activities (2.20 vs. 2.02, *p* < 0.05). Higher number of risk dietary, PA, and sedentary behaviors were all significantly correlated with higher BMI (dietary: r = 0.064; PA: r = 0.099; sedentary: r = 0.161; *p* < 0.001 for all) and body weight (dietary: r = 0.124; PA: r = 0.128; sedentary: r = 0.222; *p* < 0.001 for all). Risk sedentary behaviors was a significant risk factor for overweight/obesity (Adjusted Odds Ratio AOR = 1.30, 95% Confidence Interval CI 1.11–1.52). Obesity and unhealthy lifestyle behaviors remain a concern among Chinese adolescents. These results provide an update on the factors contributing to overweight/obesity among adolescents and call for efforts to address obesity among adolescents.

## 1. Introduction

Proper nutrition, adequate physical activity (PA), and healthy body weight are important for optimal health and chronic disease prevention. Good health practices have been associated with a reduced risk of obesity, cardiovascular diseases, type 2 diabetes, and other metabolic diseases [1]. Adolescence is a critical stage for growth and development along with habit formation. Establishing healthy dietary and PA behaviors in addition to maintaining a healthy weight early in life is a vital public health strategy for promoting lifelong overall health and well-being.

Childhood and adolescent obesity have been serious global health problems. In 2016, 50 (95%Crl 24–89) million girls and 74 (95%Crl 39–125) million boys were obese worldwide [2]. In China, the prevalence of overweight and obesity among children and adolescents aged 6–17, has increased from 16.0% to 19.0% in five years according to the Report on Nutrition and Chronic Disease Status of Chinese Residents (2020) [3].

Many adolescents do not eat a healthy diet and are not physically active at levels needed to maintain proper health. In the United States, teenagers consume fruit about 0.9 times per day and vegetables about 1.1 times per day as of 2017 [4]. In the same study, only 7.1% of high school students met federal intake recommendations for fruits, and 2.0% met these recommendations for vegetables [4]. In European adolescents aged 12.5–17.5, milk and dairy intakes were about two-thirds of the recommended amount, the fruit and vegetable intakes were about half of the recommended amount, while the intakes of meats and sweets were above the recommendations [5]. In China, the sales of sugar-sweetened beverages (SSB) have been increasing every year. The consumption rate of sugar-sweetened milk beverages and beverages for children and adolescents was 35% and 25% in the past five years, which were significantly higher than that of adults according to the Scientific Research Report on Dietary Guidelines for Chinese Residents (2021) [6]. Additionally, fried foods, sweet beverages, and smoked foods are all popular foods among adolescents [6].

The WHO and many countries have set physical activity recommendations for children and youth; unfortunately, these recommendations are not always met [7,8]. In a large-scale Health Behavior in School-aged Children (HBSC) 2010 study, samples from 37 countries, doing 60 min (1 h) or more of moderate-to-vigorous intensity physical activity (MVPA) each day was the most lacking health behavior for 15 years old girls (only met by 9.4% girls) and in boys, the percentage was 18.9%. Moreover, the amount of PA decreased as the age increased from 11 to 15 years old [8]. In the United States, the majority of adolescents (81.8%) did not meet the recommended amount of PA [9]. In a Malaysia study, more than half of the youth (13–14 years old) had low physical activity [10]. In a 2016 China Youth study, about 30% of primary, junior middle, and junior high school students met MVPA recommendations [11].

Independently of PA, sedentary behaviors, e.g., screen time, have wide-ranging adverse health impacts. For example, screen time was associated with quality of life, depression and anxiety among adolescents [12,13]. Children and adolescents spend a lot of time watching or using screens nowadays, including smartphones, tablets, gaming consoles, TVs, and computers. In the HBSC survey, 81.7% of 15 years old girls had two hours or more of screen time every day and in boys 87.2% did [8]. On average, children ages 8–12 in the United States spend four to six hours a day in screen time, and teens spend up to 9 h [14]. In a 2010–2012 China National Nutrition and Health Survey (CNNHS) study, children aged 6–17 years spent 2.9 h per day on sedentary behaviors and 85.8% of them engaged in sedentary behaviors for more than two hours a day [15].

China has experienced rapid social and economic changes in the past decade, and consequently, the lifestyle and health behaviors may have changed among Chinese adolescents. Data on the recent changes in dietary and PA behaviors among Chinese adolescents is lacking. To the best of our knowledge, the most recent reported dietary pattern data was collected in 2012, while the PA data was collected in 2017 [16,17]. Therefore, in this study, we used 2019 data from a Shanghai school-based Chinese adolescents’ health survey to assess the current dietary and PA behaviors and their association with body weight among adolescents in China.

## 2. Materials and Methods

### 2.1. Study Design and Participants

This 2019 cross-sectional study is a part of an ongoing school-based health behaviors cohort among Chinese adolescents in Shanghai. The current data was collected from October to December 2018 through multistage and stratified cluster random sampling. All districts in Shanghai were stratified into urban and suburb areas in the first stage and four districts were randomly selected afterwards. A total of 21 schools (11 middle schools, six high schools, and four vocational schools) were randomly selected in the second stage according to school type. A total of 14,038 questionnaires were filled out, with 12,860 being valid and included in the analysis (response rate was 91.6%). The students who were missing weight or height status (1038 cases) as well as those who completed the questionnaire too quickly (i.e., less than 240 s, 140 cases) were excluded.

*Written informed consents provided before enrollment, were obtained from all students, their guardians and school administrators. The study protocol was reviewed and approved by the Shanghai Jiaotong University Institutional Review Board (SJUPN-201703).*

### 2.2. Measures

This survey was part of the Shanghai Youth Health Behavior Survey, which was modified from the Youth Risk Behavior Surveillance System (YRBSS) questionnaire of the American CDC [18] and Shanghai Adolescent Health-related behavior survey questionnaire developed by Shanghai CDC. The online survey was self-administered anonymously and independently through an online survey platform (https://www.wjx.cn/, accessed on 10 December 2018) under the guidance of trained research personnel in the computer room with no teachers present. The whole questionnaire contains 96 questions and students were allowed to complete it in appropriately 15–20 min. The current study included questions including social-demographic characteristics (11 questions), dietary behaviors (eight questions), physical activity (five questions), and sedentary behaviors (four questions) and self-reported height and weight. The social-demographic characteristics included sex, date of birth, grade, accommodation, household registration, pocket money, academic achievement, parents’ occupation and education, family status, and living status. The dietary behaviors comprised frequency questions in eight areas on consumption of sugar-sweetened drinks (SSB), dessert, fried food, fruit, vegetables, breakfast, dairy, soymilk, and fast food. The physical activity behaviors included frequency questions in five areas on PA greater than 60 min daily, moderate level PA of 30 min or more daily, walking or bicycling, physical education, and afterschool activities. The sedentary behaviors addressed frequency questions in four areas on TV time, video games, the internet, and phone time.

All questions, except those assessing height and weight, were multiple choice, with five to eight mutually exclusive response options and only one possible answer per question. Based on previous reports [19], current scientific guidelines for different behaviors [20,21,22] and actual assessments [4,23], for each behavior question, a dichotomous variable was created, and responses were collapsed into either “0” or “1” [Table 1].

For each behavior domain (dietary, physical activity, and sedentary behavior), the number of risk behaviors (those coded as “1”) was summed up as a total number of risk behaviors. In total, there were eight dietary risk behaviors, five PA risk behaviors, and four sedentary risk behaviors. Based on the median of numbers of risk behaviors in each behavior domain (diet median = 2; PA median = 5; sedentary behavior median = 2), behaviors were then categorized into low (below median) and high (above median) levels.

The respondents were asked to self-report their height and weight. Body Mass Index (BMI) was calculated as weight in kilograms divided by height in meters squared (kg/m^2^). Weight status was classified as underweight, normal weight, overweight or obese using sex-specific and age- specific BMI cutoff points for Chinese children and adolescents aged 6–18 [24,25].

### 2.3. Statistical Analysis

Differences in sociodemographic characteristics by sex were tested using either the t-test for continuous variables or chi-square tests for categorical variables. For each behavior question, the frequency of consumption by sex and by weight status was assessed by chi-square tests. The means of total numbers of risk behaviors by sex and school type in three areas were compared using t-test or ANOVA followed by post-hoc tests with Bonferroni corrections. Multinomial logistic regression was used to assess the effects of lifestyle behaviors on weight status after adjusting for BMI, sex, school type, perceived weight, and other social-demographic factors. Crude odds ratios (COR) and adjusted odds ratio (AOR) and their 95% confidence interval (CI) for predictors were reported. The association between risk behaviors and weight in total or by school type and sex were assessed by Pearson’s correlation analysis. Statistical analysis was performed using SPSS 26. The significance level was set at *p* < 0.05.

## 3. Results

### 3.1. Participant Characteristics

Among all 12,860 participants between the ages of 11–18, approximately half were male students (52.1%); 22.3% classified as overweight or obese [Table 2]. Regarding school type, 43.5% were middle school students, 21.8% were high school students, and 34.7% were vocational school students. In addition, most students were living in Shanghai (94.2%), off campus (77.4%), and living with both parents (85.6%). Compared to males, female students had lower BMI (19.4 vs. 20.6), lower percentage of being overweight or obese (overweight: 9.0% vs. 18.2%; obese: 4.2% vs. 12.4%), and were more likely to be from a single parent family (8.5% vs. 6.4%) [Table 2].

### 3.2. Assessment of Dietary, Physical Activity, and Sedentary Behaviors

Among all participants, 20.3% of students had sugar-sweetened drinks ≥1 time per day; 12.1% had eaten dessert ≥1 time per day; 4.7% had fried food ≥1 time per day; while 47.2% of students had eaten fruit <1 time per day; and 25.9% had eaten vegetables <1 time per day [Table 3]. In addition, 24.3% had not eaten breakfast on all seven days of the week; 57.6% had not had dairy or soy milk on all seven days; and 44.9% had eaten fast foods at least one day of the week.

The frequency of most dietary behaviors varied by sex and body weight. Compared to males, female students less frequently consumed SSB (17.4% vs. 23.1%) and fried food (4.3% vs. 5.1%), but ate fruit (57.3% vs. 48.7%) and vegetables (77.1% vs. 71.4%) more frequently (*p* < 0.0001 for all except *p* = 0.03 for fried food). On the other hand, female students consumed dairy or soymilk less frequently (39.2% vs. 45.4%) and more frequently ate dessert (14.7% vs. 9.8%) and fast food (47.4% vs. 42.7%) than males (*p* < 0.0001 for all). Compared to normal weight students, students who were obese more frequently consumed SSB (26.2% vs. 19.5%) while they less frequently consumed fruit (50.6% vs. 53.9%), breakfast (71.5% vs. 76.4%), dairy or soy milk (39.5% vs. 42.3%) as well as dessert (7.7% vs. 13.4%) (*p* < 0.0001 for all except *p* = 0.001 for milk). Meanwhile, students who were underweight less frequently consumed fruit (48.3% vs. 53.9%), vegetables (69.2% vs. 74.8%), breakfast (74.2% vs. 76.4%) and dairy or soymilk (40.7% vs. 42.3%) (*p* < 0.0001 for all except *p* = 0.001 for milk).

Regarding PA, among all participants, less than one-quarter of students (23.2%) had 60 min or longer PA each day for seven days in a week [Table 3]. Less than one-fifth students (16.5%) had 30 min or longer moderate PA each day for 7 days a week. About one-quarter of students (24.3%) either biked or walked for 30 min or longer each day for seven days a week. In addition, about one-quarter of students (25.4%) had more than five physical education classes per week and only 15.5% of students participated in after school PA at least 5 days a week. Compared to males, female students were less frequently engaged in the above mentioned PAs (*p* < 0.0001 for all) except for physical education classes (*p* > 0.05). Compared to normal weight students, students who were obese were less frequently engaged in 60 min or more daily PA (obese 18.9% vs. normal 23.7%, *p* = 0.003) and physical education classes (obese 23.0% vs. normal 26.4%, *p* < 0.0001). There were no differences in 30 min of moderate PA, 30 min walk or bike, or after school PAs (*p* > 0.05).

Regarding sedentary behaviors, among all participants, about half of the students had one or more hours of TV or video time, video games, internet, or phone time every day (TV or video time: 54.2%; video games: 47.6%; internet: 51.6%; phone: 58.2%) [Table 3]. Compared to males, female students were less frequently to watch TV/videos (54.1% vs. 55.7%, *p* < 0.0001) or play video games (40.2% vs. 54.3%, *p* < 0.0001). No differences in internet or phone time between female and male students were identified. Compared to normal weight students, students who were either obese or underweight were more frequently engaged in all those sedentary behaviors (TV or video time: obese 62.6% & underweight 56.3% vs. normal 52.3%; video games: obese 62.2% & underweight 50.8% vs. normal 44.4%; Internet time: obese 61.3% & underweight 52.7% vs. normal 50.5%; Phone time: obese 67.5% & underweight 59.9% vs. normal 57.0%; *p* < 0.0001 for all).

**Figure 1:**
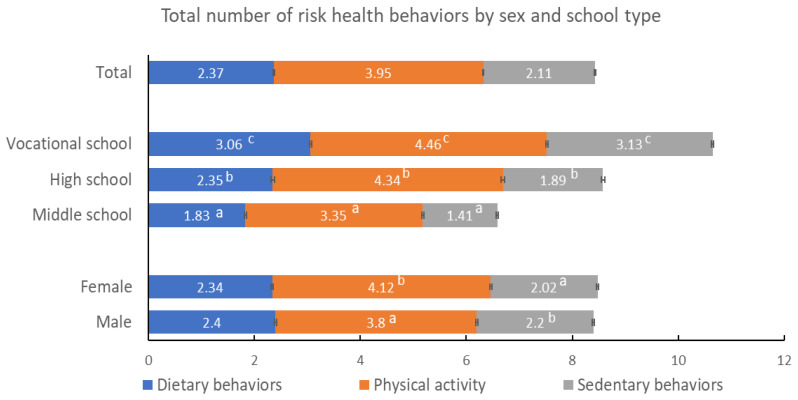
Total number of risk health behaviors by sex and school type. Lower case numbers indicate differences between sex or among school type for each lifestyle behavior (Specifically, between sex, ^a^ denotes the sex with fewer numbers of risk health behaviors and ^b^ denotes the sex with more numbers. Among school types, ^a^ denotes the school type with lowest number of risk health behavior, ^b^ denotes the school type with middle number of risk health behavior, and ^c^ denotes the school type with highest number).

Numbers of dietary risk, PA, and sedentary behaviors were reported in total and by sex and school type [Figure 1]. The average risk numbers were 2.37 out of eight dietary behaviors; 3.95 out of five PA behaviors; and 2.11 out of four sedentary behaviors. Compared to male students, female students reported more numbers of risk PA behaviors (4.12 vs. 3.80, *p* < 0.0001), less numbers of risk sedentary behaviors (2.02 vs. 2.20, *p* < 0.0001), and insignificant numbers of risk dietary behaviors (2.34 vs. 2.40, *p* > 0.05). Among school types, for each risk health behavior, vocational school students consistently had the highest number of risk behaviors, followed by high school students, and then followed by middle school students [Figure 1], *p* < 0.0001 for all.

### 3.3. Factors Associated with Weight Status in All Participants and by Sex

A higher number of risk behaviors was associated with either being underweight or being overweight/obese in the study [Table 4]. Specifically, in all participants, when having a higher number of risk PA behaviors, (i.e., less active) the odds of being underweight were higher than being more active (AOR = 1.24, 95%CI 1.10–1.40). When having a higher number of sedentary risk behaviors, the odds of being overweight or obese was 1.21 times (95%CI 1.10–1.34) higher than having a lower number of risk behaviors.

In male students, risk PA behaviors and risk sedentary behaviors had the same patterns as in all participants, i.e., higher number of risk PA behaviors had higher odds of being underweight (AOR = 1.30, 95%CI 1.11–1.52) and higher number of risk sedentary behaviors had higher odds of being overweight/obese (AOR = 1.23, 95%CI 1.09–1.39).

In female students, higher number of risk dietary behaviors had lower odds of being overweight/obese (AOR = 0.83, 95%CI 0.71, 0.98) while higher number of risk sedentary behaviors had higher odds of being overweight/obese (AOR = 1.19, 95%CI 1.00–1.41).

### 3.4. Correlations between Risk Health Behaviors and Weight

Higher number of risk dietary behaviors, PA behaviors, and sedentary behaviors were all significantly correlated with higher BMI (dietary: r = 0.064; PA: r = 0.099; sedentary: r = 0.161; *p* < 0.001 for all) and body weight (dietary: r = 0.124; PA: r = 0.128; sedentary: r = 0.222; *p* < 0.001 for all) [Table 5]. When participants are separated by sex, all significances remain for all variables for both males and females. When participants are separated by school type, the higher number of risk sedentary behaviors was still significantly correlated with higher BMI and higher weight in middle schoolers and high schoolers (BMI: middle school: r = 0.107, high school: r = 0.064; Weight: middle school: r = 0.134, high school r = 0.079; *p* < 0.001 for all). In addition, having a higher number of risk physical activities (i.e., less PA) was not correlated with BMI but lower weight in high schoolers and vocational schoolers (high school: r = −0.072; vocational school: r = −0.054; *p* < 0.001).

## 4. Discussion

The study aimed to assess the current prevalence of overweight/obesity, lifestyle risk behaviors, and their association with weight status among adolescents in China. The finding of a 22.3% of overweight and obesity rate, about half and one-quarter of students not having fruits and vegetables every day, respectively, less than one-quarter of students doing 60 min or more PA every day, and more than half of students spending one or more hours on any type of screen time indicate an alarming risk to the health status of adolescents in China. These findings vary by sex and school type.

The prevalence of overweight and obesity in the current study was higher than the prevalence from a 2016 adolescent cohort (17.6%), a 2018 children and adolescents cohort (15.8%), and the 2015–2020 national prevalence data of children and adolescents aged 6–17 (19.0%) [3,26,27]. This may indicate an increase in the prevalence of overweight and obesity among Chinese adolescents over the years. The difference may also be due to the age difference of participants included in different studies. Two of those studies include children. As the age category changes from children to adolescents, the prevalence increases [28]. In addition, the overweight and obese were more prevalent in urban than in rural regions in Chinese residents [27,29]. The study was conducted in Shanghai city, which is one of the most urbanized regions in China. The higher prevalence may also reflect the higher level of urbanization of the place the participants live. There was a higher prevalence of overweight and obesity in male students (30.6%) than in female students (13.2%). This was consistent with other reports on Chinese adolescents [26,27]. Girls tend to be more concerned with their weight and under-report their weights to a greater extent than boys [30]. Various factors could influence the weight status, including dietary behaviors and PA. Whether male students engaged in more of those health risk behaviors than females was tested in the current study and will be discussed below.

Unhealthy dietary behaviors have been associated with obesity and many chronic health conditions [31]. In the current study, many participants had low intakes of fruit, vegetables, dairy, or soymilk every day. On the other hand, high SSB consumption is still a concern for some students. This seems to be worse than reports from developed countries, for example compared to high school students in the United States, though the consumption of vegetable intake was more frequent, the fruit intake was less frequent and the SSB consumption was more frequent in the participants of current study [19]. Interventions focusing on increasing nutritional knowledge of food items and increasing the availability of healthy foods, etc. would be needed among adolescents. There was no significant difference in the average number of dietary risk behaviors between male and female students in the current study. However, among those eight dietary behaviors, more boys had a lower fruit and vegetable intake than girls. This is opposite to reports from some countries [4,32], but is consistent with reports from others [32]. This may be due to economic status or cultural differences in food preferences in different countries. In addition, boys more frequently consumed sugar-sweetened drinks than girls, which is consistent with the previous report [33]. These findings suggest that promoting healthy dietary habits among adolescents, especially boys, is important.

In China and internationally [21,34,35], adolescents are recommended to do 60 min (1 h) or more of moderate-to-vigorous intensity physical activity (MVPA) each day, including muscle-building or bone-strengthening activities three days each week. Unfortunately, the majority of participants do not meet the recommendations in the current study. There was a large variation in terms of the prevalence of having at least 60 min of MVPA per day in Chinese youth in previous reports [11,15,36,37]. For example, in two national survey studies, there were 30% and 34.1% of school-aged youth reported to have met the recommended 60 min MVPA in 2012 and in 2017, respectively, in China [36,37]. In another large sample of the national dataset, only 13.1% of Chinese children and adolescents (aged 9–17 years old) engaged in daily MVPA for at least 60 min in the last seven days in 2018 [15]. The variation may have reflected the different measures and the cutoffs used [37]. Due to the discrepancy, it is hard to identify a clear trend in the past years. Regardless, the low level of PA engagement by school-aged children and adolescents remains a problem in China and calls for school and community-based interventions or programs. On the other hand, in all studies, boys were more physically active than girls, demonstrating the sex difference in physical activities in the school-aged population. Compared to reports in developed countries, the PA status was comparable to the US youth regarding being physically active for a total of 60 min or more on all seven days or attending physical education class on all five days [19].

The low PA level was accompanied by more sedentary behaviors in these youth. Half or more than half of participants have spent one or more hours on any type of screen time (i.e., watching TV, playing video games, using internet or phone) every day. Two hours or less of screen viewing time is recommended in the guidelines [21]. The results indicated that most students did not meet the guidelines on sedentary behaviors.

Being more sedentary was positively associated with being overweight or obese in both boys and girls in the current study. This is consistent with many previous findings that watching TV, addictive phone use, and/or playing video games were associated with obesity in children and adolescents [38,39,40]. One of the reasons for the association between sedentary behaviors and overweight or obesity might be increased consumption of unhealthy food, snacks, or SSB accompanied with sedentary behaviors [41,42]. This could be due to the influence of commercials or mindless eating while engaging in screen time.

In addition, being less physically active was associated with a higher chance of being underweight, particularly in boys. This was supported by several previous studies in which low PA was associated with being underweight in adolescents, [43,44,45] and particularly in boys [37,38]. PA increases lean body mass and bone mass [44,46]. Low PA may result in less lean body mass and less bone mass, which contribute to thinness and being underweight. There are certain health risks associated with being underweight in adolescents e.g., malnutrition, vitamin deficiency, or psychiatric disorders [47]. Maintaining a healthy body weight through appropriate PA is important for overall health and well-being. Physical activity, particularly through strength training, can be encouraged for students who are underweight.

It was somewhat unexpected that engaging in more unhealthy dietary behaviors or engaging in less PA was not associated with being overweight or obese in the current study. Among eight unhealthy dietary behaviors included in this study, some (e.g., SSB intake, fast food intake, skipping breakfast, fried food) were reported to be positively associated with overweight or obesity in children and adolescents [48,49,50,51,52]. Some other studies, however, presented discrepant results between SSB and weight gain [53], no association between dessert intake, and weight [54], or even a reverse association between fast food intake and weight [55]. Some dietary behaviors (e.g., fruit and vegetable intake, dairy, or soymilk consumption) promote optimal health and contribute to good nutrition. Regarding to their impacts on the risk of obesity in children and adolescents, they either decrease or have no impact on the risk (e.g., fruit and vegetables) [56] or increase the risk (e.g., dairy) [57]. In this study, we counted all behaviors together. Some behaviors may have a greater impact than others, or they counteract with each other, which may result in no association with weight gain. Moreover, one study suggested that inactivity, rather than diet, could be driving the surge in obesity [58]. Regardless, both diet and PA have been recognized as important factors in preventing obesity. Eating healthy, being physically active, and reducing sedentary behaviors are recommended to prevent and treat obesity in children and adolescents [59].

Correlation analysis indicated that weight status was positively correlated with risk dietary behaviors, risk PA, and risk sedentary behaviors. This was the same for both boys and girls. In a previous study, BMI for adolescent boys was correlated with both eating habits and PA, however, adolescent females’ BMI was correlated with eating habits alone [60]. In addition, eating habits was a better predictor of BMI than PA, particularly for boys. In another study, PA was a greater influencer than eating habits for BMI in boys, while eating habits was a greater influencer than PA in girls [61]. All three lifestyle behaviors were correlated with weight status in the current study and in both boys and girls. This discrepancy might be due to the large sample size and greater analysis power of the current study than other studies. It might also be due to the different questions used to assess the lifestyle behaviors.

Overall, the results reflected the rapid social-economic growth and the lifestyle changes in China. Similar to many other counties, the use of electronic devices, the internet, and the social media platforms have rapidly increased among Chinese adolescents [62]. In addition, research has revealed some striking dietary changes, such as increased consumption of edible oils, animal-source foods, and sugar-sweetened beverages. The changes in cooking and eating styles include a decrease in proportion of foods steamed, baked, or boiled and more snacking and eating away from home [63]. These changes may all contribute to the overweight and obesity of Chinese youth and increase the risk of various weight-associated chronic diseases. The Chinese government has realized the severity of this public health problem, and has set the goal of “Healthy China 2030”, developing a series of policies and initiatives including promoting lifestyle changes among Chinese youth to reach the goal [64,65,66,67,68]. Some of those policies, e.g., “exercise 60 min per day” would call for efforts from all aspects including school, parents, and students themselves. The interventions in the school setting would also be important to educate and help students raise their awareness of healthy eating and active lifestyles as well as the risk of overweight and obesity in order to slow the trend.

Our study has several strengths. This study includes a large, diverse sample of students recruited using multistage and stratified cluster random sampling that provides an adequate representation of Chinese children and adolescents. We adjusted for several confounders that may influence the studied associations. A limitation of our study is that weight and height was self-reported data. This assessment may not accurately reflect the true weight status and may underestimate the magnitude of the observed associations. Self-report measures of various health behaviors (dietary behavior, PA, and sedentary behaviors) also come with challenges (e.g., social desirability bias). We used the Chinese standards of BMI cutoff points for weight status of children and adolescents in the current study. Although the ethnic specific BMI cutoff points for obesity were recommended to be used when assessing weight status in the Chinese population by previous literature [69,70], this may make it hard to compare the results with international studies. In addition, the dichotomous cut offs used to classify the risks of dietary behaviors, PA, and sedentary behaviors were only reported by a single study before [19]. Though this aligns with the current Chinese and/or international scientific guidelines [20,21,22] and recent assessment of behaviors [4,23], the validity needs to be verified by more studies. This dichotomization might also lead to a loss of relevant information compared to classifying behaviors into more than two categories.

## 5. Conclusions

Overweight and obesity, unhealthy dietary behaviors, particularly low consumption of fruit, vegetables, and dairy or soymilk are still concerns facing Chinese children and adolescents. More importantly, most students do not meet the daily recommendations for PA or the guidelines with regard to sedentary behaviors. Overweight and obesity are multifactor diseases and are influenced by various factors at individual, environmental, and systemic levels. The results updated data on the overweight, obesity and contributing factors of Chinese adolescents and call for efforts from caregivers, schools, and Chinese society to continually work together to prevent and reduce obesity among adolescents.

## Figures and Tables

**Table 1:** Question wording, details and coding for dietary, physical activity, and sedentary behaviors.

Variable	Question	Response Options	Coding for Analysis
Dietary behaviors			
Sugar sweetened drinks	During the past 7 days, how many times did you drink sugar sweetened beverages, such as soda (Coke, Pepsi, or Sprite) or sweetened milktea?	0 time, <1 time per day, 1 time per day, 2 times per day, 3 times per day, 4 times per day, 5 or more times per day	“0” was coded for <1 time/day vs. “1” for ≥1 time/day
Dessert	During the past 7 days, how many times did you eat dessert, such as candy, chocolate, cakes?	0 time, 1 time, 2–6 times, 1 time per day, 2 or more times per day	“0” was coded for <1 time/day vs. “1” for ≥1 time/day
Fried food	During the past 7 days, how many times did you eat fried food, such as Chinese cruller, fired egg rolls, fries, chicken wings?	0 time, 1 time, 2–6 times, 1 time per day, 2 or more times per day	“0” was coded for <1 time/day vs. “1” for ≥1 time/day
Fruit	During the past 7 days, how many times did you eat fruit (do not count canned fruit)?	0 time, 1 time, 2–6 times, 1 time per day, 2 or more times per day	“0” was coded for ≥1 time/day vs. “1” for <1 time/day
Vegetables	During the past 7 days, how many times did you eat vegetables?	0 time, 1 time, 2–6 times, 1 time per day, 2 or more times per day	“0” was coded for ≥1 time/day vs. “1” for <1 time/day
Breakfast	During the past 7 days, how many times did you eat breakfast?	0 day, 1 day, 2 days, 3 days, 4 days, 5 days, 6 days, 7 days	“0” was coded for 7 days vs. “1” for <7 days
Dairy or soy milk	During the past 7 days, how many days have you drunk at least one glass of milk, yogurt or soymilk?	0 day, 1 day, 2 days, 3 days, 4 days, 5 days, 6 days, 7 days	“0” was coded for 7 days vs. “1” for <7 days
Fast food	During the past 7 days, how many days have you eaten in or ordered take-out from fast food restaurants such as McDonalds, Kentucky, Pizza Hut?	0 day, 1 day, 2 days, 3 days, 4 days, 5 days, 6 days, 7 days	“0” was coded for <1 day/week vs. “1” for ≥1 day/week
Physical activity			
Days of PA 60 min or more	During the past 7 days, on how many days were you physically active for a total of at least 60 min per day (e.g., walking, jogging, playing balls, swimming, biking, or doing housework)? (Add up all the time you spent in any kind of physical activity)	0 day, 1 day, 2 days, 3 days, 4 days, 5 days, 6 days, 7 days	“0” was coded for 7 days/week vs. “1” for <7 days/week
Moderate PA 30 min	During the past 7 days, on how many days did you do moderate physical activities for at least 30 min (physical activities that increases you heart rate and made you breath hard)?	0 day, 1 day, 2 days, 3 days, 4 days, 5 days, 6 days, 7 days	“0” was coded for 7 days/week vs. “1” for <7 days/week
Walk or bicycle 30 min	During the past 7 days, on how many days did you walk or bicycle at least 30 min consecutively (including commute between school and home)	0 day, 1 day, 2 days, 3 days, 4 days, 5 days, 6 days, 7 days	“0” was coded for 7 days/week vs. “1” for <7 days/week
Physical education	In an average week when you are in school, how many physical education (PE) classes do you attend each week?	0 class, 1 class, 2 classes, 3 classes, 4 classes, 5 or more classes	“0” was coded for ≥5 times/week vs. “1” for <5 times/week
Afterschool PA	During the past 7 days, on how many days did you attend after school sport programs (such as running, playing balls, swimming, or other sports games)?	0 day, 1 day, 2 days, 3 days, 4 days, 5 or more days	“0” was coded for 5 days/week vs. “1” for <5 days/week
Sedentary behaviors			
TV or video	During the past 7 days, on average how many hours did you spent on watching TV or videos (including media video, downloaded video, DVD)?	Never, <1 h, 1 h, 2 h, 3 h, 4 or more hours	“0” was coded for <1 h/day and “1” was coded for ≥1 h/day
Video games	During the past 7 days, on average how many hours did you play video games (including handheld games consoles, on the phone, or on computer)	Never, <1 h, 1 h, 2 h, 3 h, 4 or more hours	“0” was coded for <1 h/day and “1” was coded for ≥1 h/day
Internet	During the past 7 days, on average how many hours did you spend surfing internet?	Never, <1 h, 1 h, 2 h, 3 h, 4 or more hours	“0” was coded for <1 h/day and “1” was coded for ≥1 h/day
Phone time	During the past 7 days, on average how many hours did you use your phone?	Never, <1 h, 1 h, 2 h, 3 h, 4 or more hours	“0” was coded for <1 h/day and “1” was coded for ≥1 h/day

Abbreviation: PA (physical activity).

**Table 2:** Participants demographic characteristics *.

	*n*	Total (*n* = 12,860)	Male (*n* = 6704)	Female (*n* = 6156)	*p*-Value
Age	12,860	14.6 ± 0.02	14.6 ± 0.02	14.5 ± 0.03	0.17
Height (cm)	12,860	165.0 ± 0.08	168.8 ± 0.1	160.9 ± 0.08	<0.0001
Weight (kg)	12,860	55.2.4 ± 0.1	59.5 ± 0.2	50.5 ± 0.1	<0.0001
BMI	12,860	20.1 ± 0.04	20.6 ± 0.06	19.4 ± 0.04	<0.0001
BMI categories					<0.0001
Underweight	1505	11.7	13.5	9.7	
Normal	8487	66.0	55.8	77.1	
Overweight	1780	13.8	18.2	9.0	
Obese	1088	8.5	12.4	4.2	
School type					<0.0001
Middle school	5592	43.5	43.0	44.0	
High school	2800	21.8	19.0	24.8	
Vocational school	4468	34.7	38.0	31.2	
Living on campus					0.019
Yes	2911	22.6	21.8	23.5	
No	9949	77.4	78.2	76.5	
Monthly pin money (RMB)					<0.0001
<200	6452	50.2	51.6	48.6	
200–599	4113	32.0	30.2	34.0	
≥600	2295	17.8	18.2	17.5	
Academic performance					<0.0001
Top 25%	4313	33.5	34.8	32.2	
Middle range	5442	42.3	40.5	44.3	
Bottom 25%	2339	18.2	18.6	17.7	
Live with parents					0.012
With parents	10,017	85.6	86.1	85.0	
With mother only	712	6.1	5.4	6.8	
With father only	466	4.0	4.0	4.0	
Not with parents	510	4.4	4.5	4.2	

* Data was presented as means ± SEs or percentage. Abbreviations: cm (centimeter); kg (kilogram); BMI (body mass index); RMB (renmingbi).

**Table 3:** Percentage of students who engaged in selected risk behaviors, by sex and BMI category.

	Total	Male	Female	χ^2^ Test	Under-Weight	Normal	Over-Weight	Obese	χ^2^ Test
*n* =	12,860	6704	6156		1505	8487	1780	1088	
Dietary behaviors									
Sugar sweetened drinks				<0.0001					<0.0001
<1 time/day	79.7	76.9	82.6		78.5	80.5	79.9	73.8	
≥1 time/day	20.3	23.1	17.4		21.5	19.5	20.1	26.2	
Dessert				<0.0001					<0.0001
<1 time/day	87.9	90.2	85.3		88.4	86.6	90.7	92.3	
≥1 time/day	12.1	9.8	14.7		11.6	13.4	9.3	7.7	
Fried food				0.03					0.63
<1 time/day	95.3	94.9	95.7		95.68	95.3	95.3	94.6	
≥1 time/day	4.7	5.1	4.3		4.32	4.7	4.7	5.4	
Fruit				<0.0001					<0.0001
<1 time/day	47.2	51.3	42.7		51.7	46.1	47.2	49.4	
≥1 time/day	52.8	48.7	57.3		48.3	53.9	52.8	50.6	
Vegetables				<0.0001					<0.0001
<1 time/day	25.9	28.6	22.9		30.8	25.2	24.9	25.8	
≥1 time/day	74.1	71.4	77.1		69.2	74.8	75.1	74.2	
Breakfast				0.48					0.002
<7 d/wk *	24.3	24.6	24.1		25.8	23.6	24.0	28.5	
7 d/wk *	75.7	75.4	75.9		74.2	76.4	76.0	71.5	
Dairy or soy milk				<0.0001					0.001
<7 d/wk *	57.6	54.6	60.8		59.3	57.7	53.5	60.5	
7 d/wk *	42.4	45.4	39.2		40.7	42.3	46.5	39.5	
Fast food				<0.0001					0.06
<1 d/wk *	55.1	57.3	52.6		55.3	54.4	56.2	58.4	
≥ 1 d/wk *	44.9	42.7	47.4		44.7	45.6	43.8	41.6	
Physical activity									
Days of PA 60 min or more				<0.0001					0.003
<7 d/wk *	76.8	72.8	81.1		77.7	76.3	75.9	81.1	
7 d/wk *	23.2	27.2	18.9		22.3	23.7	24.1	18.9	
Moderate PA 30 min				<0.0001					0.26
<7 d/wk *	83.5	78.8	88.6		83.9	83.5	82.2	85.0	
7 d/wk *	16.5	21.2	11.4		16.1	16.5	17.8	15.0	
Walk or bicycle 30 min				<0.0001					0.11
<7 d/wk *	75.7	73.0	78.7		76.5	76.0	73.4	75.7	
7 d/wk *	24.3	27.0	21.3		23.5	24.0	26.6	24.3	
Physical education				0.16					<0.0001
<5 tm/wk **	74.6	74.1	75.2		78.6	73.6	74.7	77.0	
≥5 tm/wk **	25.4	25.9	24.8		21.4	26.4	25.3	23.0	
After school PA				<0.0001					0.15
<5 d/wk *	84.5	81.1	88.2		85.4	84.6	82.9	85.5	
≥5 d/wk *	15.5	18.9	11.8		14.6	15.4	17.1	14.5	
Sedentary behaviors									
TV or video				<0.0001					<0.0001
<1 h/d ***	45.9	44.3	45.9		43.7	47.7	44.7	37.4	
≥1 h/d ***	54.1	55.7	54.1		56.3	52.3	55.3	62.6	
Video games				<0.0001					<0.0001
<1 h/d ***	52.4	45.7	59.8		49.2	55.6	49.2	37.8	
≥1 h/d ***	47.6	54.3	40.2		50.8	44.4	50.8	62.2	
Internet				0.56					<0.0001
<1 h/d ***	48.4	48.2	48.7		47.3	49.5	49.9	38.7	
≥1 h/d ***	51.6	51.8	51.3		52.7	50.5	50.1	61.3	
Phone time				0.51					<0.0001
<1 h/d ***	41.8	41.5	42.1		40.1	43.0	43.2	32.5	
≥1 h/d ***	58.2	58.5	57.9		59.9	57.0	56.8	67.5	

* days/week (d/wk); ** times/week (tm/wk); *** hours/day (h/d); Abbreviation: PA (physical activity).

**Table 4:** Factors associated with weight status in all participants and by sex ^1,2^.

	Normal			Underweight			Overweight/Obese	
	*n*	%	%	COR (95%CI)	AOR (95%CI) ^1^	%	COR (95%CI)	AOR (95%CI) ^1^
All participants								
Risk dietary								
Low (≤2)	7214	56.7	52.6	1	1	56.1	1	1
High (>2)	5646	43.3	47.4	1.18 (1.06, 1.32) **	1.05 (0.93, 1.18)	43.9	1.03 (0.94, 1.12)	0.93 (0.85, 1.02)
Risk PA								
Low (<5)	6383	50.4	45.0	1	1	49.9	1	1
High (=5)	6477	49.6	55.0	1.24 (1.11, 1.39) ***	1.24 (1.10, 1.40) ***	50.1	1.02 (0.94, 1.11)	1.07 (0.98, 1.18)
Risk sedentary								
Low (≤2)	6566	53.2	47.9	1	1	46.3	1	1
High (>2)	6294	46.8	52.1	1.16 (1.04, 1.30) **	1.03 (0.91, 1.17)	53.7	1.25 (1.15, 1.37) ***	1.21 (1.10, 1.34) ***
Male								
Risk dietary								
Low (≤2)	3687	55.9	51.7	1	1	54.8	1	1
High (>2)	3017	44.1	48.3	1.19 (1.03, 1.37) *	1.10 (0.94, 1.29)	45.2	1.05 (0.94, 1.16)	0.99 (0.88, 1.11)
Risk PA								
Low (<5)	3543	54.5	47.9	1	1	52.0	1	1
High (=5)	3161	45.5	52.1	1.30 (1.13, 1.51) ***	1.30 (1.11, 1.52) ***	48.0	1.10 (0.99, 1.23)	1.09 (0.97, 1.22)
Risk sedentary								
Low (≤2)	3274	51.3	45.9	1	1	45.6	1	1
High (>2)	3430	48.7	54.1	1.24 (1.13, 1.40) **	1.13 (0.95, 1.33)	54.4	1.26 (1.13, 1.40) ***	1.23 (1.09, 1.39) ***
Female								
Risk dietary								
Low (≤2)	3527	57.3	54.1	1	1	59.3	1	1
High (>2)	2629	42.7	45.9	1.14 (0.96, 1.35)	1.00 (0.83, 1.19)	40.7	0.92 (0.79, 1.07)	0.83 (0.71, 0.98) *
Risk PA								
Low (<5)	2840	47.1	40.5	1	1	44.5	1	1
High (=5)	3316	52.9	59.5	1.31 (1.10, 1.55) **	1.15 (0.95, 1.39)	55.5	1.11 (0.96, 1.29)	1.05 (0.89, 1.24)
Risk sedentary								
Low (≤2)	3292	54.7	50.9	1	1	48.3	1	1
High (>2)	2864	45.3	49.1	1.16 (0.98, 1.38)	0.92 (0.75, 1.12)	51.7	1.29 (1.11, 1.50) ***	1.19 (1.00, 1.41) *

^1^ For all participants: adjusted for sex, school type, lodging, monthly pin money, and academic performance. For male and female: adjusted for school type, lodging, monthly pin money, and academic performance. ^2^ COR: Crude Odds Ratio; AOR: Adjusted Odds Ratio; PA (physical activity) * *p* < 0.05; ** *p* < 0.01; *** *p* < 0.001.

**Table 5:** Correlations between undesired dietary, physical activities, sedentary behaviors, and weight status, by school type and sex ^1^.

r	Total	Sex		School Type		
		Male	Female	Middle School	High School	Vocational School
*n* =	12,860	6704	6156	5592	2800	4468
BMI						
Risk dietary behavior	0.064 ***	0.079 ***	0.037 **	−0.001	−0.027	−0.025
Risk PA	0.099 ***	0.108 ***	0.138 ***	0.015	−0.029	−0.004
Risk sedentary behaviors	0.161 ***	0.143 ***	0.175 ***	0.107 ***	0.064 ***	0.029
Weight						
Risk dietary behavior	0.124 ***	0.154 ***	0.080 ***	0.015	−0.010	−0.001
Risk PA	0.128 ***	0.171 ***	0.182 ***	0.002	−0.072 ***	−0.054 ***
Risk sedentary behaviors	0.222 ***	0.229 ***	0.207 ***	0.134 ***	0.079 ***	0.021

^1^ Data reported was the correlation coefficient as denoted as r ** *p* < 0.01; *** *p* < 0.001. Abbreviations: r (correlation coefficient); BMI (body mass index); PA (physical activity).

## Data Availability

The data presented in this study are available on request from authors.
